# Review of Current Achievements in Dendrimers and Nanomaterials for Potential Detection and Remediation of Chemical, Biological, Radiological and Nuclear Contamination—Integration with Artificial Intelligence and Remote Sensing Technologies

**DOI:** 10.3390/nano15181395

**Published:** 2025-09-10

**Authors:** Agnieszka Gonciarz, Robert Pich, Krzysztof A. Bogdanowicz, Witalis Pellowski, Jacek Miedziak, Sebastian Lalik, Marcin Szczepaniak, Monika Marzec, Agnieszka Iwan

**Affiliations:** 1 Faculty of Security and Safety Research, General Tadeusz Kosciuszko Military University of Land Forces, Czajkowskiego 109, 51-147 Wrocław, Poland; robert.pich@awl.edu.pl (R.P.); witalis.pellowski@awl.edu.pl (W.P.); jacek.miedziak@awl.edu.pl (J.M.); marcin.szczepaniak@awl.edu.pl (M.S.); 2 Military Institute of Engineer Technology, Obornicka 136, 50-961 Wrocław, Poland; bogdanowicz@witi.wroc.pl; 3 Institute of Physics, Jagiellonian University, Łojasiewicza 11, 30-348 Kraków, Poland; sebastian.lalik@uj.edu.pl (S.L.); monika.marzec@uj.edu.pl (M.M.)

**Keywords:** nanomaterials, dendrimers, CBRN, safety engineering

## Abstract

Current scientific and technological developments indicate that the need for dendrimers and nanomaterials should be taken into account in aspects such as the detection and remediation of chemical, biological, radiological and nuclear (CBRN) contamination. To evaluate the benefits of dendrimers in CBRN contamination, different characterization methods, toxicological evaluation, and recyclability must be used. The aim of this article is to systematize knowledge about selected nanomaterials and dendrimers as well as chemical, biological, radiological and nuclear (CBRN) hazards in accordance with the principles of green chemistry, engineering, technology and environmental safety. So far, many review articles on dendrimers and nanomaterials have focused on biomedical applications or environmental remediation. In this article, we discuss this topic in more detail, especially in relation to the integration of dendrimers with artificial intelligence and remote sensing technologies. We highlight interdisciplinary synergies—artificial intelligence for smarter design and remote sensing for deployment—that could bridge the gap between nanoscale innovation and real CBRN countermeasures.

## 1. Introduction

Nanomaterials offer new approaches and opportunities compared to traditional remediation methods and detection technologies, as evidenced by their promising performance in removing inorganic and organic contaminants, sensitivity and specific interactions. Such nanomaterials include carbon nanotubes, fullerenes, graphene, aerogels, zeolites, dendrimers, self-assembling monolayers, various types of nanoparticles and nanofibers, and quantum dots. The basic characteristics of nanomaterials that determine their wide applications include their high specific surface area (defined as S = F/m, where F is the surface area and m is mass), high activity, catalytic surface area, adsorption, tendency to agglomerate, chemically diverse composition, and natural or synthetic origin. For example, noble metal nanoparticles (Ag, Au, and Pt), carbon-based nanomaterials, nanocomposites, enzyme-based biosensors and cyclodextrin derivatives could be useful in the future for remediation evaluation and contaminant detection. It is known that in the context of unconventional CBRN threats, nanosystems and nano-devices pose a risk of “dual” use. Nanomaterials can be used as a resource for humanity, providing new tools for protection and decontamination from CBRN agents, and as powerful tools for detecting new threats. However, at the same time, such nanosystems can become an unpredictable and uncontrollable means of mass destruction through the design of innovative and more effective aggressive agents or the large-scale production of precursors for deliberately toxic systems. We should also not forget about the growing role of artificial intelligence (AI), which can be helpful but can also pose a threat in the development of nanotechnology and CBRN detection and remediation processes.

For nanomaterials to be considered as durable and reliable tools for purification methods, it is necessary to focus on adapting their synthesis for large-scale production. Currently, the synthesis of nanomaterials is based on methods such as combustion, plasma use, liquid-phase processes, chemical precipitation, sol–gel method, mechanical processes, mechanochemical synthesis, high-energy ball milling, chemical vapor deposition and laser ablation. In the large-scale production of nanomaterials, a greener and more economical approach should be taken into account while studying the environmental impact in order to prevent environmental pollution. It is necessary to use safe technology, which involves discerning the properties of materials and by-products and the use of appropriate catalysts and solvents, to prevent environmental pollution (ensuring safety for humans). In addition, since the efficacy of nanomaterials is usually studied at the laboratory scale, an evaluation of results under environmental conditions is essential to ensure their effective use. The synthesis and use of nanomaterials should be consistent at both micro and macro levels with the REACH (Registration, Evaluation, Authorisation of Chemicals) system, the SVHC (Substances of Very High Concern) Candidate List and the BAT (Best Available Techniques) concept, which refers to the set of technical devices, methods of operation and organizational solutions that are best suited to prevent, reduce or eliminate pollutants (which have been applied at both scales and do not entail excessive costs to adapt to conditions and demand).

Our previous work was devoted to the study of the properties of four generations of thiophosphoryl–PMMH dendrimers for their use in the detection and remediation of CBRN contamination. On the basis of dielectric studies, we have shown that three out of four generations of thiophosphoryl–PMMH dendrimers are an alternative to linear dielectric polymers (the dielectric constant and low dielectric losses are similar for these materials). A coupled technique (a thermographic camera and multichannel potentiostat–galvanostat) was used to evaluate the feasibility of using PMMH dendrimers as an organic conductive layer in a detection system for detecting CBRN agents. We have shown that the heat distribution was uniform without any structural defects for all four generation of dendrimers studied [1]. Moreover, we have been working on covering photovoltaic (PV) panels with materials with self-cleaning properties (including nano-TiO_2_ and TiO_2_-Ag) with photocatalytic properties [2]. Our special method of covering the PV panels with a layer of nano-TiO_2_ ensured the ability of the panels to self-clean without the use of detergents or special cleaning techniques, i.e., it reduced CO_2_ emissions and additionally contributed to environmental cleaning. In turn, reducing environmental pollution contributes to the reduction in acid rain. TiO_2_ with various amounts of Ag were obtained by us by the sol–gel method [3]. We also have experience in implementing such nanomaterials as [6, 6] phenyl-C61-butyric acid methyl ester (PCBM), graphene, and carbon nanotubes into solar cells or flexible layers in order to produce biodegradable electrodes [4,5,6,7].

The presented research was developed as part of the research project entitled “Specialist set for collecting forensic traces of crime from places contaminated CBRN agents” (CBRN-BOX). During the project, a special CBRN protective suit was developed for work in CBRN-contaminated areas, a mobile decontamination chamber (tents) was designed, and preventive protection and first aid packages were developed for use in places at risk of CBRN agents. The project also aimed to determine and estimate the probability and effect of undesirable events during the performance of official tasks in places contaminated with CBRN agents.

**Figure 1:**
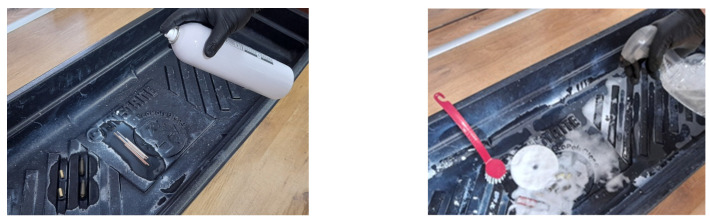
Decontamination of forensic trace media using powder and foams containing substances that neutralize (remove) CBRN agents.

Another objective of the project was to develop procedures for safe work with CBRN agents contaminated with forensic traces. Despite a number of known decontamination methods, the problem of the possibility of “destroying” key features of revealed forensic traces during decontamination has still not been solved. Currently, experts are also unable to work with CBRN agents contaminated with forensic traces. Therefore, the possibility of using nanomaterials in both detection and decontamination is being considered, especially in the case of sensitive forensic traces, e.g., biological traces. The experience gained during the project shows that the techniques used for the detection or decontamination of CBRN agents with the use of nanomaterials are an alternative to classic solutions, which are not useful due to the potentially destructive nature of the revealed forensic traces (Figure 1).

The main goal of the CBRN-BOX project was to develop a specialized kit for the exploration of forensic traces from sites contaminated with CBRN agents, consisting of a mobile decontamination chamber with an accompanying tent, special protective clothing, and preventive protection and first aid kits to support forensic operations. The project also aimed to develop a procedure and method for the on-scene decontamination of recovered data carriers serving as substrates for forensic traces, which are then sent for laboratory tests by experts. Practice indicates that most decontamination methods are not useful due to the “destruction” of key features of the forensic traces. Currently, experts are unable to work with contaminated traces. It is also impossible to store contaminated forensic traces. Based on a literature review and our preliminary studies [1] regarding the use of thiophosphoryl–PMMH dendrimers, we believe that dendrimers can be used as

(a) CBRN binding materials that can facilitate their detection or physical removal from surfaces or environment;
(b) Nanocarriers for neutralizing agents that are released upon contact with the contaminant, providing rapid protection.

Therefore, as part of the ongoing project, we are considering the possibility of using dendrimers for both detection and decontamination, particularly in the case of damage-sensitive forensic traces, such as biological traces. CBRN detection or decontamination techniques using dendrimers are an alternative to traditional solutions, which are not useful due to the potentially destructive nature of the forensic traces. To our knowledge, this topic has not been previously explored in the literature.

The aim of this work is to systematize knowledge in the field of selected nanomaterials and dendrimers and chemical–biological–radiological–nuclear (CBRN) threats in accordance with the principles of green chemistry, engineering, technology and ecological safety. To our knowledge, this is the first study on the synergy of nanomaterials and CBRN risks.

This review provides a comprehensive synthesis of knowledge on dendrimers and related nanomaterials for CBRN detection and remediation while highlighting their integration into emerging digital technologies. Beyond cataloging material classes, morphology, and functionalization strategies, this overview emphasizes AI-driven design and optimization, illustrating how machine learning can predict dendrimer architectures, surface chemistry, and hybrid compositions to improve specificity, sensitivity, and multifunctionality. By combining experimental datasets with predictive models, this approach accelerates the discovery of high-performance materials while minimizing trial-and-error synthesis.

Additionally, this review discusses the potential of remote sensing and field-deployable sensor networks, showing how dendrimer-based nanosensors can be coupled with UAVs, environmental monitoring platforms, and real-time spectral analysis to achieve scalable, on-site detection of chemical, biological, radiological, and nuclear agents. The framework presented situates dendrimers within a broader digital–material ecosystem, bridging nanoscale innovation with translational applications. Collectively, these insights highlight the synergistic potential of combining nanomaterial science, AI, and remote sensing to achieve next-generation CBRN countermeasures, providing a roadmap for both researchers and practitioners in the field.

## 2. Methods and Techniques Used for Material Detection and Remediation

The ability to rapidly detect and effectively neutralize CBRN materials is crucial for defense, public health, and emergency response. While significant progress has been made in the development of detection and remediation technologies in recent years, there are still numerous challenges in their effectiveness, portability, and integration into crisis management systems. Modern chemical detectors use advanced spectroscopic techniques such as mass spectrometry, infrared spectroscopy, and Raman spectroscopy to the enable precise identification of chemical substances at the molecular level. For example, a lightweight chemical detector based on ion mobility spectrometry can be an effective tool in CBRN units. The detection of pathogens and biological toxins relies on methods such as polymer chain reaction (PCR), immunoanalysis, and biosensors. New biological detection systems are designed to rapidly identify threats in the field, maintaining high sensitivity and specificity. Modern radiological detection systems use advanced Geiger-Müller counters, gamma radiation spectrometers, and artificial intelligence-based technologies to precisely locate radiation sources and assess the threat. In response to the need to minimize risk to personnel, remote sensing technologies such as unmanned ground vehicles (UGVs) and aerial vehicles (UAVs) equipped with chemical, biological, and radiological sensors are being developed. These systems enable remote threat mapping and sampling for analysis.

Modern methods of chemical neutralization include the use of electrochemical and photocatalytic decontamination and nanotechnology. These methods enable the effective removal of chemical contaminants from surfaces and the environment, often without the use of water, which is important in harsh field conditions. For biological materials, methods such as enzymatic deactivation, the use of biocides and UV radiation are used. New technologies, such as the production of nanomaterials with antimicrobial properties, offer the potential to inactivate pathogens quickly and efficiently. The neutralization of radiological materials involves their removal or decontamination with specialized equipment, such as robots with manipulators or remote monitoring systems. The use of artificial intelligence to analyze data from detectors allows for the precise location and characteristics of the threat. In response to the need for rapid field response, mobile remediation systems are being developed, such as vehicles equipped with decontamination technologies, that enable the rapid removal of contamination over large areas.

Chemical agents are toxic substances designed to harm or kill people through their physiological effects. These agents can be used as chemical weapons (CWs) or in industrial applications. They are classified based on their effects on the body, including nerve agents, blister agents, choking agents, and blood agents. Table 1 presents a classification of the main chemical warfare agents.

The Chemical Weapons Convention (CWC), overseen by the Organization for the Prohibition of Chemical Weapons (OPCW), imposes very strict and unambiguous restrictions on field research involving the use of chemical warfare agents (CWs). The guiding principle is a total ban on such research, with very narrow and strictly controlled exceptions. A detailed discussion of these restrictions is provided in Table 2.

In summary, the CWC effectively prohibits the open, extensive field research with chemical warfare agents, which was common during the Cold War. Any permitted activities are extremely narrow, strictly regulated, and overseen by international bodies. Literature reports treat simulants as substances imitating the action of warfare agents by analogy to functional groups—which are centers of chemical interactions—but devoid of toxic properties.

**Figure 2:**
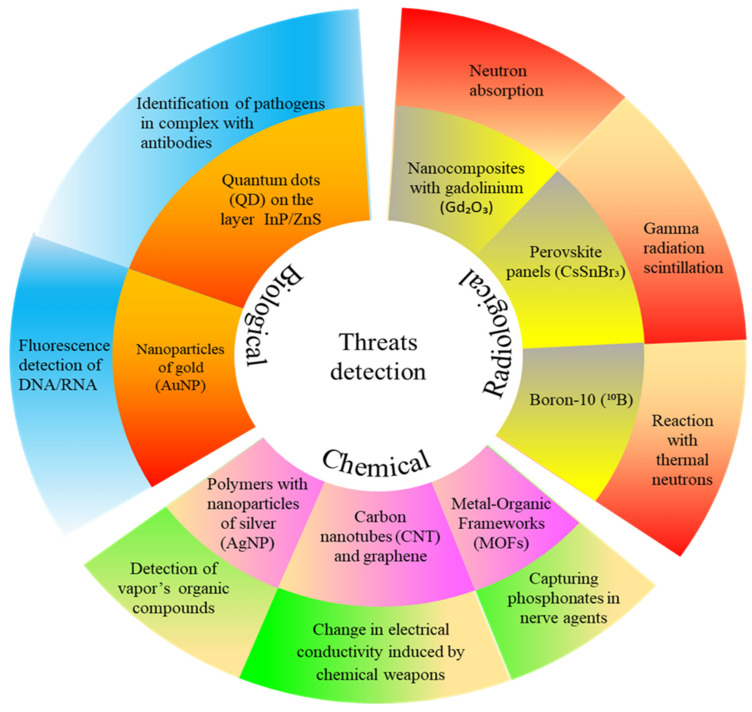
Examples of nanomaterials and technologies used in CBRN detection.

Current methodologies for the detection of chemical, biological, radiological and nuclear (CBRN) agents are based on a wide variety of principles, from simple colorimetric tests to complex spectrophotometric techniques for off-site detection. Nanotechnologies can also provide some innovations in this field. Nanomaterials exhibit unique physicochemical properties that enable their use in the detection and remediation of environmental contaminants. The design of sensors, biosensors or other devices capable of detecting or neutralizing these contaminants focuses on improving features such as efficiency, sensitivity and detection time. In the literature, derivatives of noble metal nanoparticles, carbon-based nanomaterials, nanocomposites, enzymatic biosensors and cyclodextrin derivatives are particularly intensively studied in this respect. Figure 2 presents examples of nanomaterials and technologies used in CBRN detection.

CBRN decontamination is mainly based on the physical removal, dissolution, or chemical destruction of the contaminant [8]. Novel techniques based on nanosystems can provide catalytic active solids with acidic, alkaline or ORP functionalities that increase the rate of the decomposition of harmful contaminants, even at room temperature. These techniques can provide an alternative process to conventional thermal destruction and incineration to reduce CBRN weapons [9].

Such nanomaterials, which are often inorganic oxides, such as TiO_2_, MgO, CaO, ZnO, Al_2_O_3_, MnO_2_, and transition metal-containing nanoporous silica, are very promising, acting as “reactive sorbents” or catalyst supports for the destruction of chemical warfare agents due to their highly reactive defect sites (edges, corner and lattice planes). A commercial product Fast-Act^®^ made up of MgO and TiO_2_ has been developed by the NanoScale Corporation in order to degrade warfare agents such as sulfur mustard, soman, and VX agent (see Table 1) [10].

Wagner et al. studied the hydrolysis of VX, soman and sulfur mustard on TiO_2_ materials for the development of self-decontamination paints that can be used to protect military vehicles [11]. Recently, cerium dioxide nanoparticles have shown exciting potential for the fast degradation of organophosphorous and organochloride compounds. Janoš, Salerno and Trenque evaluated the efficiency of CeO_2_ and CeO_2_ composites in the degradation of organophosphorus nerve agents such as soman, VX and pesticides, e.g., parathion, with an emphasis on the rapid and highly efficient conversion of toxic products into non-toxic ones [12,13,14].

Dendrimers—highly branched, monodisperse macromolecules—are increasingly being utilized as templates, stabilizers, and delivery vehicles for inorganic nanoparticles (NPs). Their well-defined architecture, internal cavities, and surface functional groups make them uniquely suited for controlling nanoparticle size, dispersion, and biocompatibility. When combined with inorganic nanoparticles, dendrimers can yield multifunctional nanocomposites with enhanced properties for biomedical, catalytic, electronic, and environmental applications (see Table 3).

In summary, dendrimers represent a versatile scaffold for the synthesis, stabilization and functionalization of inorganic nanoparticles with the following key characteristics:

➢ Gold nanoparticles (Au NPs) in multiple shapes are leading candidates in cancer detection and therapy due to their low cytotoxicity and tuneable optical properties.
Silver nanoparticles (Ag NPs) are notable for their antibacterial and conductive applications.
Selenium nanoparticles (Se NPs) have emerged as effective nanocarriers for drug and gene delivery with antioxidant and anticancer potential.
Metal oxide NPs (e.g., Fe_3_O_4_, ZnO, TiO_2_) are critical for imaging, catalysis, and therapeutic functions.
Fullerenes and derivatives represent carbon-based nanomaterials with unique biomedical applications in PDT, antioxidation, and antiviral therapy.

Dendrimers, inorganic nanoparticles, and carbon-based nanomaterials together form a powerful toolkit in the field of nanomedicine, diagnostics, and advanced nanotechnology fields.

Another interesting class of compounds are bimetallic nanoparticles (BNPs), which represent a class of nanomaterials composed of two different metallic elements combined at the nanoscale. Their importance lies in the fact that combining two metals often leads to synergistic properties not present in the individual monometallic counterparts. By carefully tuning the size, shape, composition, and arrangement of the two metals, scientists can design BNPs with optimized catalytic, optical, magnetic, and biomedical functionalities (see Table 4).

Overall, dendrimer-encapsulated bimetallic nanoparticles (DENs) provide a highly tunable platform at the intersection of nanoscience, catalysis and biomedicine. Their precise structure, synergistic metal interactions and dendrimer functionality create unique opportunities for next-generation applications in catalysis, imaging, drug delivery and energy technologies. They are among the most promising nanomaterials currently being studied due to their role in combining basic science and practical innovation.

Moreover, we would like to highlight that in our last article [1], we made a report evaluating PMMH dendrimers against leading CBRN detection and remediation platforms, focusing on performance indicators such as limit of detection (LOD), response time, and operational stability. The evaluation identifies areas of advantages and weakness over alternatives such as MOF composites (ZIF-8, UiO-66-NH_2_), metal-oxide nanofibers, and enzyme-based gels.

## 3. Application of Dendrimers

### 3.1. Beneficial Applications of Dendrimers in CBRN

**Figure 3:**
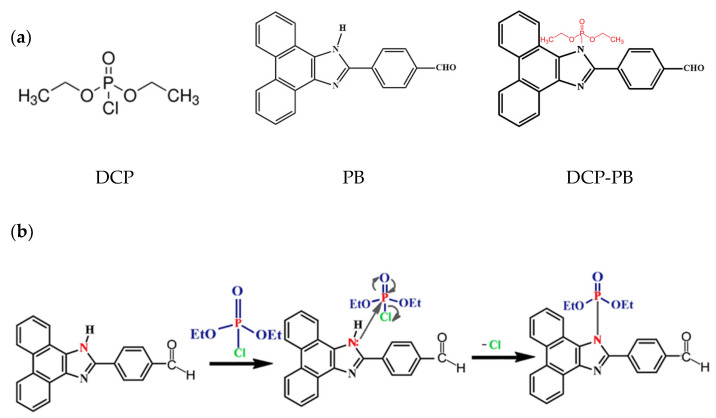
Chemical structure of DCP, PB and DCP-PB (**a**) and the probable sensing mechanism of PB with target analyte DCP (**b**).

An example of studies on the use of dendrimers with fluorescent tags for the detection of sarin is the use of phenanthrenimidazole-based phosphorus for the selective identification of the sarin simulant diethylchlorophosphate (DCP). DCP was used to simulate sarin because it is less hazardous than real nerve agents while still having similar reactivity. This study describes the development of a 4-(1H-phenanthrenimidazol-2-yl)benzaldehyde (PB) luminophore that selectively recognizes the sarin simulant, DCP, with a detection limit in the micromolar range. Upon the addition of the DCP to PB solution, a change in fluorescence from cyan to green was observed. Upon the addition of triethylamine to the PB-DCP solution, the fluorescence returned to its original state, allowing for multiple uses of the sensor. The chemical structure of DCP, PB and DCP-PB is presented in Figure 3a. Tests using paper strips and RGB color analysis using a smartphone were also conducted, confirming the practical utility of the PB sensor for rapid DCP detection (Figure 3b) [15].

Another example is the development of a new fluorophore with an active N-H vibration in the benzimidazole group for the sensitive detection of gaseous sarin. The interactions between the nucleophilic fluorine atom in sarin and the electrophilic safety atom in the benzimidazole group of the fluorophore restrict the N-H vibration, leading to the occurrence of fluorescence. Based on this, the experimental and theoretical detection limits of gaseous sarin reach 50 and 4.8 ppb, respectively. Additionally, the co-assemblies of this fluorophore with other donor-acceptor fluorophores trigger differential responses to gaseous sarin in general, with specific interferences, indicating its potential application under appropriate conditions [16].

**Figure 4:**
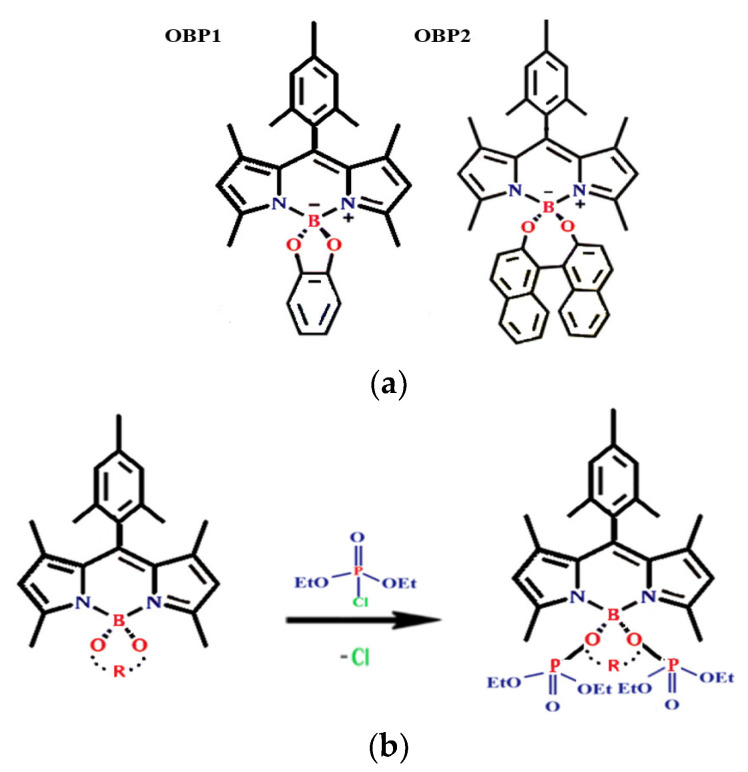
Chemical structure of OBP1 and OBP2, spiro-4,4-diaryloxy-BODIPY derivatives (**a**) and the probable sensing mechanism for the reactive 4,4-dialkoxy-BODIPY derivatives to detect DCP (**b**).

An example of the use of thiophosphoryl–PMMH dendrimers in CBRN contamination is also the prototype of a compact fluorescent sarin detector based on reactive derivatives of 4,4-diaryloxy-BODIPY [1,17]. The chemical structure of two high-performance fluorescent materials, spiro-4,4-diaryloxy-BODIPY derivatives, denoted as OBP1 and OBP2, is shown in Figure 4a. The OBP1 and OBP2 emission reactions are highly sensitive to the presence of DCP, showing a significant change in fluorescence color to a light green color within 5 min. A prototype of a compact device with a laminated sensor design was also developed, which provided the device with high efficiency, sensitivity and durability in detecting sarin at the submillimolar level in a solution environment [18]. The probable sensing mechanism for the reactive 4,4-dialkoxy-BODIPY derivatives to detect DCP is shown in Figure 4b.

### 3.2. Other Application of Dendrimers

Dendrimers have so far found application in medicine, in particular as drug delivery carriers. There are several applications for dendrimers. The first one is dendrimers as a cellular drug delivery carrier. This means that the commonly available painkiller ibuprofen enters the cell in 3 h. Dendrimers, on the other hand, cause ibuprofen to enter the cell within one hour. This shows that dendrimers can effectively transport a complex drug into the interior of the cell. The second application of dendrimers is their use as solubility enhancers. Due to their hydrophobic and hydrophilic layer properties, dendrimers enhance the solubility of poorly soluble drugs by forming covalent or non-covalent complexes with molecular drug molecules and hydrophobes [19].

In turn, Yasmin et al. [17], in view of the rapid increase in dengue (which has been a real health problem for many years), proposed an analytical method using a biofunctionalized sensor with a tapered optical fiber (TOF) integrated with a polyamide dendrimer (PAMAM) to detect dengue E protein (DENV II E). Tapered optical fiber sensors follow today’s trends in sensor designs, promoting detection system solutions with exceptional sensitivity and selectivity.

Another example of the use of dendrimers is in the delivery of drugs to the eye, which involves the use of PAMAM dendrimers with carboxylic or hydroxyl surface groups. This solution increases the availability of pilocarpine, thereby creating a non-irritating, biocompatible and sterile environment for administering the drug to the eyes. They are also used in oral drug delivery, where a significant advantage is reduced fluctuation in the plasma level of the drug due to controlled drug delivery systems, as the drug is slowly released from the dose in a continuous manner and maintains a constant blood level. However, in addition to the advantages, there are also disadvantages such as poor solubility in aqueous solutions and poor penetration through intestinal membranes.

There are many examples of the use of dendrimers in drug delivery. The most common of them have been described above. However, examples of the use of dendrimers can also be found in the delivery of drugs with targeted and controlled release, which reduces the cytotoxicity of the anticancer drug and increases uptake by cancer cells, e.g., dendrimers in the administration of drugs to the lungs and percutaneously [19]. We can even say that dendrimers play a key role in the controlled release of drugs, i.e., they have the ability to deliver drugs for a longer period of time at a controlled rate. This usually involves implanting a specially designed polymer directly into the organ or system that is affected. Dendrimers provide a lower polydispersity index and a controlled, well-defined size and structure that can be easily modified and can alter the chemical properties of the system. In addition, dendrimers have increased permeability and a greater retention effect that allows them to target cancer cells more quickly [20].

In addition to drug delivery applications, dendrimers can be used in cosmetics, particularly in cosmetic products that are used as ophthalmic carriers. L’Oreal has obtained a patent for the use of dendrimers in the production of mascaras and nail polishes. Unilever holds a patent for the use of dendrimers in the production of sprays, gels and lotions. In addition to the cosmetics industry, they are also used to purify water contaminated with toxic metals and organic and inorganic substances [19].

Dendrimers are nanostructures used as carriers of anticancer drugs, which, thanks to the possibility of functionalization, can be precisely targeted at tumor foci. Their biocompatibility and unique properties make them useful not only in drug delivery, but also in the diagnosis and transdermal administration of active substances. Traditional cancer therapies often affect both healthy and diseased cells, leading to serious side effects. Dendrimers, on the other hand, can selectively deliver drugs or genetic material without causing toxicity, actively targeting cancer cells, e.g., by using antibodies directed against specific antigens. By incorporating different components into their structure—such as fluorescent dyes, DNA sequences or targeting agents—dendrimers can act as therapeutic molecules, combining diagnostics and therapy in a single system. This allows for the simultaneous monitoring of the treatment and control of drug dosing. The use of dendrimers as DNA carriers or anticancer drugs can significantly increase the effectiveness of anticancer therapy. Their structure allows for modification, which allows for increased bioavailability and improved treatment effectiveness. Progress in nano-oncological research means that dendrimers may play a key role as modern therapeutic tools in the fight against cancer in the future [21].

Over the past two decades, dendrimer chemistry has made significant advances, enabling the creation of complex structures with controlled size and composition. As demonstrated above, dendrimers have great potential as elements of hybrid materials, combining their structure with inorganic nanoparticles. Their unique physicochemical properties make them promising candidates for sensor applications including colorimetry, fluorescence, electrochemistry, and surface-enhanced Raman spectroscopy (SERS). Although the number of such hybrid materials is growing rapidly, the range of dendrimers used is still relatively narrow—PAMAM dominates, mainly due to its availability and the simplicity of its synthesis. However, the use of other types of dendrimers can bring a new quality to sensor design, providing knowledge on the relationship between structure, activity and physicochemical properties. Although PAMAM inorganic materials show great potential, their actual implementation and commercialization remain limited. Further applied research could change this, especially if we consider the possibility of the selective binding of analytes and improved colloidal stability. To date, most research has focused on hybrids with a single inorganic phase. However, dendrimers offer the possibility of integrating various inorganic nanoparticles with complementary functions into a single structure, which may result in the development of modern, multifunctional detection platforms with high sensitivity and selectivity. Although the use of dendrimers in SERS systems is relatively poorly understood, their potential in this area warrants an in-depth review of existing developments. Technological advances in the field of Raman spectroscopy, including the development of portable devices, open up new possibilities for practical applications. Dendrimers are ideally suited for the formulation of substrates that meet the specific requirements for the detection of analytes in trace amounts. Their ability to form multifunctional structures, especially those combining magnetic and plasmonic properties, makes them extremely suitable for systems that require sample preconcentration and selective detection. With the ability to modify the surface of plasmonic nanoparticles, dendrimers also allow high analytical selectivity to be achieved even in complex environments. This suggests that magnetoplasmonic structures based on dendrimers can be effectively used both for the capture of chemical compounds and their subsequent detection, e.g., by SERS [22].

### 3.3. The Role of Dendrimers as Novel Nanocarrier in Cancer Therapy, Including Passive Targeting and Receptor-Targeted Drug Delivery for a Deeper Understanding of CBRN Contamination

Dendrimers belong to a new group of compounds increasingly used in many scientific fields. Their unique properties like their nanoscale uniform size, high degree of branching, polyvalence, water solubility, and interaction with cell membranes and various active drug molecules as well as the characteristics of their internal structures and cavities, make dendrimers excellent candidates for drug delivery systems (DDSs) [23]. Not only dendrimers but also many others types of nanoparticles, such as polymer micelles, biodegradable polymer nanoparticles, nanocomposites, fullerenes, nanocapsules, nanogels, nanoliposomes, solid lipid nanoparticles and metal nanoparticles, have been used as drug delivery systems [24,25]. The goal of drug delivery systems is to encapsulate bioactive substances within a carrier structure, thereby protecting them during transport to the target site and ensuring effective penetration across the cell membrane [26]. The challenge facing drug delivery systems is to release therapeutic agents at the right time and place, in a repeatable and safe manner. The benefits of many drugs cannot be exploited because of their toxicity, poor solubility or stability problems. Dendrimers are promising candidates for improving the solubility and to diminish the toxicity of drugs with a narrow therapeutic index [27]. They are considered as miracle carriers and are being investigated for the severe diseases such as cancer, AIDS, tuberculosis [28]. Dendrimers are also widely studied as DDS for their potential use in anti-inflammatory, antiviral, antibiotic and cardiovascular disease therapies, but the primary area of research is the use of dendrimers as ideal carriers for various anticancer drugs [29]. Dendrimer–drug interactions can be divided into two main types:

(a) Physical interactions—based on the inclusion of the active drug molecule into the central structure of the dendrimer through non-covalent associations, hydrogen bonding, or hydrophobic or electrostatic interactions [30,31].
(b) Chemical interactions—involving the covalent conjugation of drugs with the functional end groups of dendrimers [32,33].

The encapsulation of active substances within the dendrimer improves drug stability and can ensure its controlled delivery to cancer cells. Dendrimers have been highlighted in numerous encapsulation studies of various anticancer substances, such as methotrexate (MTX), camptothecin (CPT), 5-fluorouracil (5-FU), doxorubicin free base (DOX) and paclitaxel (PTX). These molecules have two major drawbacks: low hydro solubility and high nonspecific toxicity. Hence, the use of dendrimers is a promising strategy [34,35,36,37,38].

Polyamidoamine (PAMAM) and poly(propylene imine) (PPI) dendrimers are examples of a versatile and reproducible type of nanocarrier that can be drug-loaded and modified by attaching specific (target-dependent) ligands that recognize receptors that are overexpressed on cancer cells. There are numerous studies in the literature in which drugs recommended for cancer have been conjugated with poly(amidoamine) dendrimers. For example, doxorubicin (DOX) is used to treat lung cancer and brain tumors (glioblastoma). It is conjugated to fifth-generation PAMAM (G4) dendrimers, where conjugation takes place by acylhydrazone bonds on the surface of the dendrimer. The benefit lies in the increase in therapeutic efficacy and specificity of action in lung cancer by the direct action of the pH-controlled DEXTRIMER DOX-PEG-PAMAM [39,40]. In turn, Satsangi showed that paclitaxel (PTX) conjugated with a PAMAM G4 dendrimer through a glycine–phenylalanine–leucine–glycine peptide, a linker for the indication of breast cancer, increases specificity and cytotoxicity compared to the PTX molecule alone [41]. As part of the gastrointestinal cancer study, Dichwalkar studied PTX conjugated to the dendrimer PAMAM-G4-DHA inoculated with omega-3 fatty acids. In the case of this type of conjugation, an increase in pharmacological activity in upper gastrointestinal cancer was demonstrated compared to the cytotoxic molecule alone [42]. Sunitinib, used in renal neoplasm, has been conjugated with an NH2-PAMAM-G3 dendrimer through a platinum (II)-based binding system, obtaining the targeting of the active molecule to neoplastic renal tissue [43].

Poly(propylene imine) dendrimers are generally characterized by the presence of primary amine terminal groups and tertiary propylene amines inside the PPI structure. The main mechanism by which these dendrimers act is to increase the solubility of the conjugated drug through electrostatic interactions [44]. Melphalan, used in breast cancer, is an example of a dendrimer conjugate obtained by combining the dendrimer of PPI with folic acid. This increased both the biocompatibility of the active molecule due to the protection of cationic folate groups and the inhibition of tumor development and increased survival, especially G4 and G5. However, the build-up of these cationic dendrimers in a generation is associated with higher toxicity [45]. Gorzkiewicz synthesized a A PPI G5 dendrimer conjugate loaded with DOX and dextran, which showed improved absorption on the A549 cancer cell line, as well as a sustained release profile of the active drug molecule, and at the same time decreased hemolytic activity [46]. In the literature, there are studies in which methotrexate (MTX) is conjugated to a dendrimer to release the active molecule to the desired site of action, improving the efficiency of methotrexate targeting and transport to cancer cells. Tekade has synthesized PPI G5 dendrimers modified with folate and loaded with MTX and retinoic acid, designed to transport the active drug specifically to cancer cells. In their studies, they showed an improvement in the effectiveness of methotrexate targeting and transport to cancer cells [47].

Apart from anticancer drugs, PAMAM dendrimers with amine-terminated surface groups might be potential carriers for non-steroidal anti-inflammatory drugs (NSAIDs) which possess carboxyl groups. Nonsteroidal anti-inflammatory drugs that have been successfully encapsulated in PAMAM or complexed with it include aspirin, indomethacin, flurbiprofen, ketoprofen, ibuprofen, diclofenac and naproxen [48,49]. Of the active substances mentioned, the interactions in the dendrimer–ibuprofen system have been most extensively studied to date. Due to the presence of hydroxyl groups, the latter interacts electrostatically with the surface amino groups of PAMAM dendrimers. Milhem et al. demonstrated that 40 ibuprofen molecules interact with the G4.0 PAMAM dendrimer at pH = 10.5 [50]. Kolhe et al., however, obtained 78 ibuprofen molecules bound to the same dendrimer. Due to the 64 surface amine groups in the PAMAM G4 structure, it was assumed that some of the drug molecules interact with internal tertiary amines, confirming the possibility of the simultaneous encapsulation and surface immobilization of active substances [51].

Koc and Senel synthesized a PAMAM dendrimer with enhanced efficacy in NSAID delivery to the site of action by introducing a propylene oxide (PPO) residue into the central structure of the PAMAM dendrimer. These dendrimers were then conjugated to the spirocles of ketoprofen, ibuprofen and diflunisal. It was also shown that the solubility of these drugs increased with dendrimer production, due to the increased size of the nucleus and because the internal structure of the PP-PAMAM dendrimer corresponds to the optimal drug interaction, compared to the simple PAMAM dendrimer [52]. The PAMAM dendrimer was also tested for the release of antibacterial drugs. When these dendrimers interact with water-soluble antibiotics, an improvement in the antibacterial properties can be observed. For example, Cheng showed that fluoroquinolones (perifloxacin and prulifloxacin) conjugated to PAMAM G4 dendrimers with ethylene–diamine surface groups (64 NH_2_ groups) showed a significant increase in antimicrobial activity and water solubility [53].

A number of studies have also been conducted describing the possibility of encapsulating not only drug molecules but also genetic material, proteins, targeting factors and dyes in the dendrymer structure by encapsulation, complexation or conjugation [54].

The above constitutes a small review of the literature on the use of nanomaterials, including dendrimers, which have proven valuable in both diagnosis and therapy, due to their ability to improve solubility, absorption, bioavailability, and targeted distribution.

Based on a literature review and our preliminary studies regarding the use of thiophosphoryl–PMMH dendrimers [1], we believe that dendrimers could be used as binding materials for CBRN substances, which would facilitate their detection or physical removal from surfaces or the environment, or as nanocarriers for neutralizing agents that would be released upon contact with the contaminant, providing rapid protection. The results obtained in biomedicine so far encourage the pursuit of new studies into the use of dendrimers for CBRN detection and remediation. They can also provide valuable information for delivery for a deeper understanding of CBRN contamination.

## 4. Methods Used to Characterize Dendrimers

Various analytical techniques are used to study the chemical composition, morphology, shape, polydispersity, homogeneity, synthesis, conjugation, reaction rate, molecular weight, structural defects, and purity of dendrimers. These include spectroscopic methods (NMR, EPR, UV-Vis, CD, IR, Raman, dielectric spectroscopy), scattering techniques, microscopic methods, chromatographic techniques, electrical techniques, and rheological/physical property analysis. Applications of spectroscopic techniques in the characterization of dendrimers are presented in Table 5 [55,56,57,58,59,60,61,62,63,64,65,66,67,68,69,70,71,72,73,74,75,76,77,78,79,80,81].

**Table 5:** Applications of spectroscopic techniques in dendrimer characterization.

Analytical Techniques	Applications	Ref.
UV-Vis Spectroscopy	proof of synthesis—characteristic curves exhibit the specific maximum absorption peaks	[55]
	conjugation (surface modification)—due to characteristic absorption maximum or bathochromic shift	[56]
	reaction rate	[57]
IR Spectroscopy	proof of synthesis—characteristic peaks corresponding to functional groups	[58]
	conjugation (surface modification)—due to shifts in characteristic peaks corresponding to functional groups	[59]
	proof of synthesis progress by appearance–disappearance–reappearance of characteristics peaks	[60]
NMR Spectroscopy	synthesis of dendrimers—characteristic peaks in the spectra	[61]
	conjugation chemistry—shielding/deshielding effects shifts in peaks	[62]
	hydrodynamic radii–NMR pulse–field gradient spin–echo	[62]
	number of protons—intensity of peaks and integral value	[63]
	conformational changes	[63]
	mobility of group	[64]
Mass Spectrometry	determining the molecular weight	[65]
	detailed studies of structural defects in dendrimers	[66]
	determination of the polydispersity and purity of dendrimers	[67]
Raman Spectroscopy	structure	[68]
	liberation of terminal groups in dendrimers	[69]
	interaction between dendrimer with lipid membranes	[70]
Fluorescence Spectroscopy	interaction between the drug and dendrimers	[71]
	the size and shape of the molecules	[72]
	peripherally modification	[72]
Atomic Force Spectroscopy	characterize the structure	[73]
	interaction of the different dendrimer therapeutics with a lipid bilayer, behavior of the dendrimer agents	[73]
X-ray Photoelectron Spectroscopy	elemental composition	[74]
	empirical formula	[75]
	chemical state	[76]
	thickness of one or more thin layered dendrimers	[77]
X-ray Absorption Spectroscopy	structural information	[78]
	local geometric and electronic structures	[78]
EPR Spectroscopy	determining the numbers	[79]
	distributions of numbers	[80]
	spatial distribution of molecule	[81]
Dielectric Spectroscopy	determining of dielectric processes	[1]
	determining of dielectric constant and loss	[1]
	determining of specific conductivity	[1]
IR-thermal technique	terminal imaging	[1]
	current–temperature relationship	[1]
	heat distribution and structure defect information	[1]

## 5. Toxicological Assessment

The rapid development of nanotechnology and the widespread use of nanomaterials in industry, agriculture and medicine have led to research into the harmfulness of nanoparticles to the human body. The literature reports on the toxicity of nanoparticles vary widely. However, most researchers believe that their large surface area and small size are the main causes of their toxicity [82]. Recent studies have focused on understanding the mechanisms of nanoparticle toxicity and identifying the factors that contribute to their adverse effects [83]. The key determinants of nanoparticle toxicity are primarily the size, shape, surface area, surface charge, solubility and chemical composition of nanoparticles. In addition, nanoparticles’ interactions with biological systems such as proteins, enzymes and DNA can affect their toxicity. It has been shown that nanoparticles can enter the human body through inhalation, ingestion and skin contact, causing damage to cells, tissues and organs, including the lungs, liver, kidneys and brain. There are many studies in the literature on the effects of nanoparticles on the respiratory system, nervous system, endocrine system, immune system and reproductive system, as well as their relationship with the occurrence and development of cancer [84].

Dendrimers, as polymeric nanomaterials, are currently being studied for biomedical applications such as medical imaging, gene therapy and tissue-targeted therapy. Higher-generation (size) dendrimers are of interest and have been widely studied for their ability to carry drugs. However, the use of dendrimers in the biological system is limited due to the inherent toxicity associated with them. Dendrimers and other cationic macromolecular delivery systems interact non-specifically with the negatively charged biological membrane. This non-selective interaction of the cationic delivery system causes membrane disruption through nanohole formation, membrane thinning and membrane erosion. Consequently, this leads to cytosolic enzyme leakage and cell death. There are various toxicities of dendrimers, including cytotoxicity, hemolytic toxicity, and hematological toxicity. Unfortunately, most studies to date have focused on the toxicity of dendrimers in cell cultures, with fewer studies investigating in vivo toxicity.

**Figure 5:**
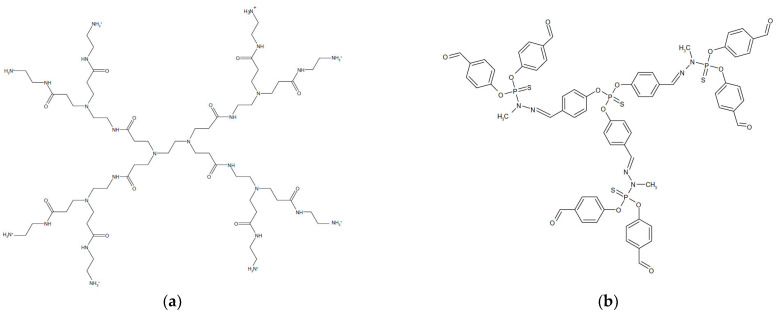
Chemical structure of (**a**) generation 1 polyamidoamine (PAMAM) dendrimer and (**b**) thiophosphoryl–phenoxymethyl (methylhydrazono) 1.5 generation dendrimer.

Pryor et al. [85] investigated the toxicity of several polyamidoamine (PAMAM) dendrimers (Figure 5a) and thiophosphoryl dendrimers (Figure 5b), which differed in generation and surface charge, using embryonic zebrafish (Danio rerio) as a model vertebrate. They showed that higher-generation cationic dendrimers were more toxic than lower-generation anionic or neutral dendrimers with the same core composition. PAMAM dendrimers caused significant morbidity and mortality as the generation decreased [85]. The observed results did not confirm the trends previously observed in cell culture studies [86], which showed that an increase in dendrimer generation is associated with an increase in toxicity. However, no significant adverse effects were observed with the suite of thiophosphoryl dendrimers studied.

Malik et al. [87] showed that regardless of the internal structure of the repeat units, cationic dendrimers are generally hemolytic and cytotoxic, depending on the molecular weight (generation) and the number of surface groups. Conversely, anionic dendrimers were neither lytic nor cytotoxic over a wide range of concentrations, and studies showed no evidence of in vivo toxicity when injected repeatedly into mice. Agashe and Kolhatkar have shown that dendrimers such as PPI (poly (propylenenoimine)), PAMAM (polyamidoamine), and PLL (poly-L-lysine) exert significant cytotoxicity in vitro due to their surface cationic groups [88].

Chen et al. [89] reported on the cytotoxicity of cationic melamine dendrimers with surface groups such as amine, guanidine, carboxylate, sulfone, causing cell lysis. A variety of physicochemical properties such as size distribution, electrostatics, surface area, overall morphology and aggregation can significantly influence the physiological interactions between nanomaterials and dendrimers and target biological areas. As such, it is critical to fine-tune these properties to ensure that the biological target is met safely.

## 6. Recycling of Nanomaterials

The recycling and disposal of nanomaterials is challenging due to their small size and complex composition. Various methods of nanoparticle separation are used in the recycling of nanowaste, including (i) centrifugation, (ii) solvent evaporation, (iii) magnetic separation, (iv) the use of pH/heat-sensitive materials, (v) molecular antisolvents, and (vi) nanostructured colloidal solvents. One of the physical techniques for separating nanomaterials from water is the use of a magnetic field. This method has gained recognition for its simplicity, affordability, high efficiency, repeatability, low power requirements, and applicability to both batch processes and large-scale (industrial) operations.

**Figure 6:**
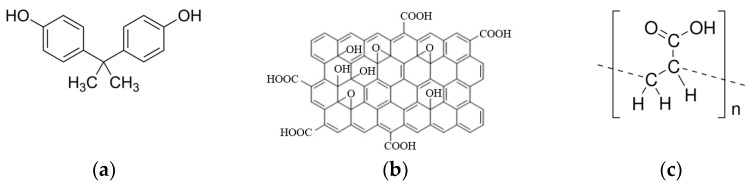
Chemical structure of (**a**) bisphenol A, (**b**) graphene oxide and (**c**) polyacrylic acid.

For example, Grass et al. [90] placed a commercial neodymium magnet on the outside of a colloid containing cobalt nanoparticles and separated the nanoparticles from the water in less than 20 s. Zhang et al. removed bisphenol A (Figure 6a) from water using graphene oxide (GO, Figure 6b) composites GO/Fe3O4-modified with polyacrylic acid (PAA, Figure 6c). PAA/GO/Fe_3_O_4_ nanocomposites, due to their large surface area, excellent complexing capacity and superparamagnetism, were used as nanoadsorbents for the recyclable removal of Cu^2+^, Cd^2+^ and Pb^2+^ ions from aqueous solutions. Moreover, PAA/GO/Fe_3_O_4_ nanocomposites are very easy to separate and recycle due to the superparamagnetism of Fe_3_O_4_ [91].

Another variant of magnetic separation is magnetic flocculation, which combines flocculation and magnetism. This involves adding a flocculation agent to the magnetic nanoparticles that allows them to agglomerate so that the magnet can attract the nanoparticles faster and more efficiently during separation. This method also allows for increased recycling efficiency (a higher percentage of recovered nanoparticles) [92].

A common technique for physically separating nanomaterials from water is the centrifugation process. This method uses high-speed centrifugation to separate nanoparticles from a liquid medium based on size, density, and sedimentation rate. Rajesh et al. [93] developed trimetallic Al-Cd-Mn nanoparticles for the photocatalytic removal of dye in water using visible light. The valuable trimetallic nanoparticles can be recycled through a simple centrifugation process and are ready for reuse. In contrast, Gargari et al. [94] used silica/polyvinylimidazole core-envelope nanoparticles (SiO_2_/PVI/H_2_PO_4_-NPs) to remove samarium and dysprosium ions from water due to their unique characteristics, such as their large surface area and increased adsorption and desorption rates. The core-shell nanoparticles were recovered by centrifugation at 14,000 rpm for 20 min. An alternative, cost-effective and highly efficient approach to the recovery and reuse of nanomaterials after water treatment involves immobilizing them on a solid structure such as polymer, ceramic or metal.

Chemical methods for the recycling/disposal of nanomaterials include the following:

- Cloud Point Extraction (CPE), a liquid–liquid extraction process in which a surfactant is added to produce micelles that, at a certain temperature, separate the target nanoparticles from the aqueous solution, allowing them to be easily recovered. Nazar et al. [95] used CPE using Triton X-114/Triton X-100 nonionic surfactants to recycle Ag and Pd nanoparticles.
- Crystal growth.
- A chemical-based recovery technique. Zhuang et al. [96] recovered Sn nanoparticles by adding NaOH as a mineralizer to form an amorphous Sn compound that could be recycled by dissolving the formed solid in an acid.

A major disadvantage of chemical separation compared to physical nanoparticle separation is the requirement of additional chemicals that can be harmful in themselves and need to be separated from water. In contrast, the advantages of physical methods such as filtration, magnetic separation and coagulation are as follows: (i) they do not require potentially harmful chemicals, (ii) they involve fewer processing steps, which is relevant for large-scale applications, and (iii) they allow for easier automation and implementation of AI, which enable a more sustainable environment using fewer human resources in such dangerous environments.

## 7. Discussion

Dendrimers, with their precisely controlled branching architecture, monodispersity and easy surface functionalization, and other advanced nanomaterials (such as metal nanoparticles, Metal-Organic Frameworks (MOFs), carbon nanotubes and graphene) are on the verge of a revolution in the detection and decontamination of chemical, biological, radiological and nuclear (CBRN) contaminants. Their unique physicochemical properties enable the design of intelligent, highly sensitive and specific platforms capable of operating in extreme conditions. They are characterized by advanced detection and effective decontamination, which are described below.

### 7.1. Advanced Sensing

- Multiplexing and selectivity: Future systems will use dendrimers as scaffolds to simultaneously immobilize multiple receptors (e.g., aptamers, antibodies, chemical ligands) or fluorescent/electrochemical sensors. This will enable multiple threats (e.g., neurotoxin and pathogen) to be detected simultaneously in real time from a single environmental or clinical sample. The functionalization of dendrimer end groups will provide remarkable selectivity by minimizing matrix interference.
- Ultra-high sensitivity: Hybrids of dendrimer nanoparticles and metal (e.g., Au, Ag) or carbon nanomaterials amplify the detection signals (e.g., through the SERS effect —Surface-Enhanced Raman Scattering, fluorescence enhancement). This will enable the detection of single molecules of chemical toxins (e.g., VX, sarin) or single cells of pathogens (e.g., anthrax, plague) at the attomolar level.
- Intelligent platforms: Nanosensors will be developed that respond to a specific target by clearly changing the signal (e.g., color, fluorescence, electrical conductivity). Dendrimers equipped with photochromic or chelating groups will act as “logic gates”, activating only in the presence of a specific combination of threats.
- Radiological/nuclear detection: Dendrimers functionalized with strong chelators (e.g., DOTA, catechol derivatives) capture radioactive metal ions (U, Pu, Cs, Sr, Am). In combination with scintillation nanoparticles or semiconductor detectors, they will create portable, highly sensitive systems for mapping radioisotope contamination.

### 7.2. Effective Decontamination (Remediation)

- Catalytic decomposition: Dendrimers will serve as ideal carriers for catalytic nanoparticles (e.g., Pd, Pt, TiO_2_) or enzymes (e.g., oxidases, hydrolases). This architecture will provide high catalyst dispersion, substrate availability and stability, allowing for rapid and complete decomposition of chemical warfare agents (e.g., G and V series, mustard gas) or toxic pesticides at the contamination site.
- Adsorption and separation: Dendrimer–MOFs or dendrimer–graphene hybrids will have a large specific surface area and numerous active sites (dendrimer functional groups). This will enable ultra-tight adsorption of a wide range of hazards: toxic chemicals (including heavy metals), pathogens (via electrostatic/van der Waals interactions) and even radioisotopes (via chelation). These materials will be regenerated in situ (e.g., by pH changes, rinsing with saline solutions or irradiation with light).
- Biological neutralization: Dendrimers with a controlled charge (cationic) and size directly inactivate viruses and bacteria, destabilizing their membranes. Functionalization with antimicrobial groups (e.g., chitosan derivatives, AMP peptides) will create a strong synergistic effect against resistant biological pathogens.
- Surface decontamination: Dendrimer-based gel and foam reagents capable of adhering to complex surfaces (concrete, metal, and fabric) will be developed. They will simultaneously absorb, catalyze degradation and biologically inactivate a wide range of CBRN agents, facilitating the decontamination of areas and infrastructure.

As shown above, these materials will face challenges in the future. Despite their enormous potential, key challenges include toxicological studies of the long-term environmental and human health effects of nanomaterials, the scalable and cost-effective production of complex nanostructures, and ensuring stability and reliability in extreme battlefield or disaster conditions. The integration of these nanoplatforms with Lab-on-a-Chip, robotics and artificial intelligence for real-time data analysis will be key to creating the next generation of autonomous, portable and versatile CBRN systems.

**Figure 7:**
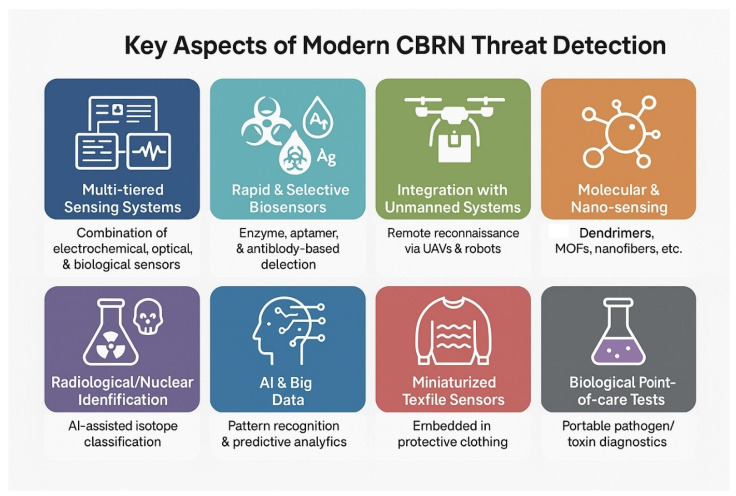
Key aspects of modern CBRN threat detection.

Nanomaterials, especially dendrimers and their hybrids, are a fundamental element of the future of CBRN technology. Their ability to be precisely engineered at the molecular level paves the way for the creation of intelligent, multifunctional platforms with unprecedented sensitivity, speed and effectiveness in neutralizing various threats. Further research to address current challenges can significantly enhance national security and global capabilities to respond to CBRN incidents. Key aspects of modern CBRN threat detection are presented in Figure 7.

The strategy of this review focused on dendrimers and nanomaterials (inorganic metals, oxides, salts, carbon fullerenes, and hybrids such as DEN) in the context of chemical, biological, radiological, and nuclear (CBRN) contaminant detection and removal. The focus was on which nanomaterials have been best proven for contaminant detection and removal, how structure–function relationships influence performance, and what barriers hinder translation (stability, contamination, cost, and regulatory issues). Primary scientific databases (Web of Science, Scopus, PubMed, Embase, IEEE Xplore, ACS, etc.), preprints, theses, gray literature (e.g., NATO, WHO, IAEA), patents, and standards were used. The scope covered the period 2000–2025, with a focus on recent work, and inclusion was limited to experimental or validated simulation studies related to CBRN contaminants or simulants, providing performance metrics and material characterization. Expected results are presented in Table 6.

Advancements in dendrimer-based nanocomposites for pollutant detection have laid a strong foundation for environmental monitoring. To build upon this progress, future research should prioritize the integration of emerging materials and technologies (see Table 7).

To advance this field, future research should focus on the following:

➢ Multifunctional nanocomposites: The development of nanocomposites that combine the advantages of dendrimers and quantum dots could lead to sensors capable of detecting a wide range of pollutants, including radioactive substances.
Sustainable synthesis methods: Adopting green nanotechnology approaches for synthesizing these materials can reduce the negative environmental impact and enhance the sustainability of pollution sensing technologies.
Integration with remote sensing platforms: Incorporating these advanced sensors into unmanned aerial and ground vehicles can facilitate real-time, large-scale environmental monitoring, improving response times to pollution events.

By focusing on the integration of quantum dots and dendrimer-based nanocomposites, future research can lead to the development of advanced, multifunctional sensors for pollution detection. A focus on sustainable synthesis methods and integration with remote sensing platforms will ensure that these technologies are both effective and environmentally friendly.

## 8. Conclusions

This article attempts to systematize knowledge in the field of selected nanomaterials and dendrimers as well as chemical–biological–radiological–nuclear (CBRN) hazards in accordance with the principles of green chemistry, engineering, technology and ecological safety. We have collected and presented examples of nanomaterials and technologies used in CBRN detection along with the benefits of dendrimers in CBRN contamination. The current applications of dendrimers have also been widely described. Research methods used to characterize dendrimers, ranging from chemical composition, morphology, shape, polydispersity, homogeneity, synthesis, conjugation, reaction rate, and molecular weight to structural defects and purity studies, were collected and described. Issues of toxicity and recycling of nanomaterials were also discussed.

Summarizing, despite technological advances, challenges remain in the detection and remediation of CBRN materials, including the following:

- The integration of detection and remediation systems: The need to develop integrated platforms for the comprehensive management of CBRN threats.
- Mobility and autonomy: The development of unmanned detection and remediation systems capable of operating in challenging terrain.
- Artificial intelligence and data analysis: The use artificial intelligence to analyze detector data for faster and more precise threat identification.
- Personnel training: The need for the ongoing training of emergency services and the military in the use of modern CBRN technologies.

Many previous reviews on dendrimers and nanomaterials have focused on biomedical or environmental remediation applications. We tried to explore the topic further. Here are some unique insights, especially with regard to the integration of dendrimers with artificial intelligence and remote sensing technologies.

➢ AI-enabled material design and optimization:
  (a) Predictive modeling: AI and machine learning can help design dendrimer architectures with tailored branching, surface groups, or hybrid compositions (e.g., dendrimer–metal oxides, DENs) for optimal binding of CBRN agents.
  (b) Data-driven synthesis: Training AI on spectral, structural, and toxicological datasets enables the rapid screening of candidate dendrimers prior to synthesis, reducing experimental trial and error.
  (c) Adaptive nanomaterials: AI-driven optimization could guide the dynamic functionalization of dendrimers for multiplexed detection (e.g., detecting chemical simulants in complex backgrounds).
Remote sensing integration for field deployments:
  (a) Nano-sensor networks: Dendrimers and DENs functionalized with optical or electrochemical reporters can be embedded into sensor arrays connected to remote platforms (e.g., UAVs, environmental monitoring stations).
  (b) Real-time detection: Coupling dendrimer-based recognition with AI-enhanced image/signal analysis (e.g., SERS spectral interpretation, fluorescence lifetime data) allows the rapid, on-site identification of chemical or biological agents.
  (c) Scalable monitoring: Remote sensing technologies could integrate dendrimer-based nanodevices into distributed networks, extending their utility from lab-scale proof-of-concepts to large-area CBRN surveillance.
Translational insight.

This overview offers a forward-looking framework that is not limited to just summarizing the properties of materials. They place dendrimers and hybrid nanomaterials in a digital and sensing ecosystem that is currently being adopted by defense, healthcare, and environmental agencies. It highlights interdisciplinary synergies—AI for smarter design, remote sensing for deployment that could bridge the gap between nanoscale innovation and real-world CBRN countermeasures.

## Figures and Tables

**Table 1:** Classification, names, NATO code and chemical structures of chemical warfare agents.

Classification	Name	Code	Chemical Structure
**nerve agents** (disrupt the nervous system by inhibiting the acetylcholinesterase enzyme, leading to the overstimulation of muscles and glands)	**sarin** (o-isopropyl methyl phosphonofluoridate)	GB	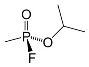
	**tabun** (ethyl *N*,*N*-dimethylphosphoramidocyanidate)	GA	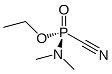
	**soman** (3,3-dimethylbutan-2-yl methylphosphonofluoridate)	GD	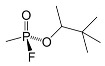
	**VX** (O-ethyl S-diisopropylaminoethylmethylphosphothioate)	VX	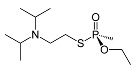
**blister agents** (cause severe skin, eye, and respiratory tract injuries by damaging tissues upon contact)	**sulfur mustard** (1-chloro-2-[(2-chloroethyl)sulfanyl] ethane)	H/HD	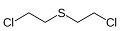
	**nitrogen mustards** (bis(2-chloroethyl)ethylamine)	HN-1, HN-2, HN-3	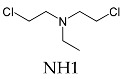 NH1
	**lewisite** (2-chloroethenyldichloroarsine)	L	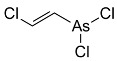
**blood agents** (disrupt the body’s ability to use oxygen, leading to suffocation at the cellular level)	**prussic acid** (hydrogen cyanide)	AC	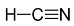
	**cyanogen chloride** (chloroformonitrile)	CK	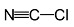
**choking agents** (cause damage to the respiratory system, leading to fluid buildup in the lungs and potentially fatal pulmonary edema)	**phosgene** (carbonyl dichloride)	CG	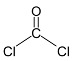
	**diphosgene** (trichloromethyl carbonochloridate)	DP	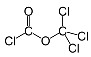
	**chloropicrin** (trichloro (nitro) methane)	PS	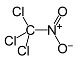
**non-lethal agents** (used as a riot control agent, tear gas, vomiting agents or sneeze gases)	**CS** ([(2-chlorophenyl)methylidene]propanedinitrile)	CS	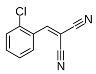
	**dibenzoxazepine** (dibenzo[*b*,*f*][1,4]oxazepine)	CR	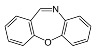
	**adamsite** (10-Chloro-5,10-dihydrophenazarsinine)	DM	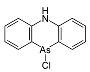
	**chloroacetophenone** (2-chloro-1-phenylethan-1-one)	CS	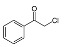
**psychological agents** (aim to temporarily disable individuals by causing confusion, hallucinations, or other mental disturbances without permanent harm)	**3-quinuclidinyl benzilate** (1-azabicyclo [2,2,2]octan-3-yl hydroxy(diphenyl) acetate)	BZ	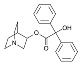
	**lysergic acid diethylamide** ((6a*R*,9*R*)-*N*,*N*-diethyl-7-methyl-4,6,6a,7,8,9-hexahydroindolo[4,3-*fg*]quinoline-9-carboxamide)	LSD-25	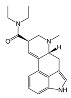
	**psilocybin** ([3-[2-(dimethylamino)ethyl]-1*H*-indol-4-yl] dihydrogen phosphate)	4-PO-DMT	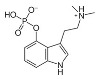

**Table 2:** The Chemical Weapons Convention restrictions.

**1. The fundamental prohibition (Article I of the convention).**
The most important provision is found in Article I, paragraph 1 (d), of the Convention, which states that each State Party adheres to the following: “Never, under any circumstances: (d) To use chemical weapons.” Since “use” within the meaning of the Convention also includes “testing,” any field research involving the actual use of CWs is considered the use of chemical weapons and is strictly prohibited.
**2. Exceptions and permitted types of research**
The Convention permits research with the use of chemical substances, but only for very specific, peaceful purposes and under strictly defined conditions. Field research with CW agents is only permitted in the following contexts: (a) Chemical defense research *What is Permitted:* States Parties are authorized to conduct research to develop protective measures against chemical weapons (e.g., test suits, filters, sensors, antidotes, decontamination methods). *Restrictions:* Scale and Quantity: Strictly minimal quantities of CW agents necessary to achieve the test objective may be used for research. Large-scale testing using large quantities of agents is prohibited. *Type of Substance:* Tests must be justified by a real threat. The use of the most dangerous agents (e.g., VX, sarin) is extremely rigorously controlled. *Location:* Tests are often conducted in closed, controlled chambers or at specialized, closed testing ranges to minimize the risk of environmental contamination. Notification: States may (and in some cases must) report their defense research programs to the OPCW, although details often remain confidential. (b) Industrial (Peaceful) research *What is Permitted:* Research, development, and testing for industrial, agricultural, research, medical, pharmaceutical, or other peaceful purposes is permitted. *Restrictions:* Type of substances: This applies primarily to so-called precursors (chemicals used to produce CW agents) or CW agents themselves in quantities and concentrations that have no military application (e.g., testing phosphate-based pesticides). *Prohibition:* No research may be aimed at developing new types of chemical weapons or improving existing ones. (c) Toxicological and medical testing *What is Permitted:* Conducting toxicological studies under controlled laboratory conditions to understand the mechanisms of action of poisons and develop treatments. *Restrictions:* These are almost exclusively in vitro (cell-based) tests or animal models. Human testing is strictly prohibited by international law and ethics.
**3. Infrastructure and site restrictions**
*Declaration and Inspections:* All facilities where chemical weapons have ever been produced or tested (so-called formerly chemical weapon-related facilities) must be declared to the OPCW. They are subject to routine and challenge inspections to confirm that they are not being used for prohibited activities. *Closed Test Ranges:* Any defensive testing may only take place in specially designated, secured, and isolated testing ranges to avoid the accidental exposure of civilians or environmental contamination.
**Summary—Key restrictions:**
*Comprehensive Combat Ban:* Any testing aimed at developing, evaluating the effectiveness, or improving chemical weapons is illegal. *Minimum Quantities:* Permitted research (defensive, peaceful) may only use the minimum necessary quantities of CW agents. Strict Purpose: The purpose of the research must fall within the scope of chemical weapon protection or the peaceful use of chemistry. *Transparency and Control:* All activities related to CW agents are subject to potential verification and inspection by the OPCW. *Environmental and Public Protection:* Test methods and locations must effectively prevent the release of hazardous substances into the environment.

**Table 3:** Short summary of dendrimers and inorganic nanoparticles.

**Inorganic nanoparticles categories: Elemental metal nanoparticles**	
Elemental metals form some of the most widely studied nanoparticles due to their tunable optical, electrical, and catalytic properties.	
Gold nanoparticles (Au NPs)	Au NPs are among the most versatile nanomaterials. They can be synthesized in diverse morphologies—nanospheres, nanorods, nanostars, nanoshells, and nanocages—each with unique surface plasmon resonance (SPR) properties. Biomedically promising: Their strong optical absorption/scattering enables biosensing, disease detection, imaging, and photothermal therapy (PTT). Au NPs show particularly strong potential in cancer diagnosis and treatment, with low cytotoxicity in vitro, making them safer candidates compared to many other metals. Surface versatility: Au NPs can be functionalized with drugs, antibodies, or nucleic acids for targeted delivery.
Silver nanoparticles (Ag NPs)	Known for high conductivity and potent antibacterial properties, Ag NPs are widely used in wound dressings, coatings, and biosensors. Their antimicrobial action is linked to the release of Ag^+^ ions and the generation of reactive oxygen species (ROS). When encapsulated within dendrimers, Ag NPs gain enhanced stability and controlled ion release, making them more biocompatible for medical uses.
Selenium nanoparticles (Se NPs)	Over the last five years, Se NPs have gained remarkable interest in drug and gene delivery. Selenium is an essential trace element, and nano-size improves its bioavailability, antioxidant activity, and reduced toxicity compared to bulk Se. Dendrimer-assisted Se NPs are now being explored as nanocarriers for chemotherapeutics, gene silencing agents, and nutraceuticals, with promising results in cancer therapy, neuroprotection, and immunomodulation.
**Metal oxide nanoparticles**	
Metal oxides are another important class of inorganic nanomaterials. Examples include ZnO, TiO_2_, Fe_3_O_4_, and CeO_2_ nanoparticles, which exhibit photocatalytic, magnetic, or antioxidant properties.	
ZnO NPs	for antimicrobial and UV-protective coatings
TiO_2_ NPs	in photocatalysis, sunscreens, and drug delivery
Fe_3_O_4_ (magnetite) NPs	in magnetic resonance imaging (MRI), targeted drug delivery, and hyperthermia cancer treatments
CeO_2_ NPs	as “nanozymes” with antioxidant enzyme-mimetic activity, promising in oxidative stress-related diseases
**Metal salts and hybrid nanostructures**	
Nanoparticles derived from salts (e.g., CaCO_3_, BaSO_4_) serve as biocompatible carriers for drugs and imaging agents. Dendrimer encapsulation helps stabilize such nanoparticles, prevents premature dissolution, and allows for surface modification with targeting ligands.	
**Carbon-based nanomaterials: Fullerenes and derivatives**	
Beyond inorganic nanoparticles, carbon nanostructures such as fullerenes (C_60_ and higher derivatives) have gained prominence. Structure: Fullerenes are hollow, cage-like molecules composed entirely of carbon atoms. Their spherical or ellipsoidal geometry, high electron affinity, and ability to generate singlet oxygen under light make them unique. Applications: Drug and gene delivery through conjugation with biomolecules. Antioxidants and radical scavengers, protecting cells from oxidative damage. Photodynamic therapy (PDT): Fullerene derivatives produce reactive oxygen species (ROS) upon light activation, selectively killing cancer cells. Antiviral and neuroprotective agents due to their ability to cross cell membranes and interact with biological macromolecules. Dendrimers are frequently employed to functionalize fullerenes, enhancing their solubility and biocompatibility, thereby expanding their medical and technological applications.	

**Table 4:** Short summary of bimetallic nanoparticles.

**1. Bimetallic nanoparticles: Structure and properties**
BNPs can exist in various structural motifs, such as - Alloyed nanoparticles: The two metals are uniformly mixed at the atomic level. - Core–shell structures: One metal forms the core while the other forms the shell. - Cluster-in-cluster or segregated morphologies: Distinct domains of each metal coexist within the nanoparticle. These structures significantly affect their - Electronic properties (due to charge transfer and orbital hybridization); - Catalytic activity (enhanced reactivity and selectivity); - Optical behavior (tunable surface plasmon resonances); - Stability and durability (synergistic stabilization effects).
**2. Dendrimer-encapsulated nanoparticles (DENs)**
DEN is a special subclass of nanomaterials in which nanoparticles are synthesized and stabilized inside the cavities of dendrimers—branched, tree-like polymers with well-defined structures. Encapsulation advantages: - Prevents aggregation, ensuring high stability; - Provides size control at the sub-2 nm scale; - Allows for precise control over metal composition; - Offers functional groups on the dendrimer surface for bioconjugation. Synthesis: DENs are typically prepared by coordinating metal ions within dendrimer cavities, followed by chemical reduction. For bimetallic DENs, either co-reduction (to form alloys) or sequential reduction (to form core–shell structures) is employed.
**3. Unique properties of bimetallic DENs**
Compared to bulk metals or monometallic DENs, bimetallic DENs exhibit - Enhanced catalytic efficiency due to synergistic metal–metal interactions; - High selectivity in chemical transformations (important in fine chemical synthesis); - Stability in physiological environments, making them attractive for biomedical uses; - Tunable optical features (plasmonic and photothermal properties).
**4. Biomedical applications**
Bimetallic DENs have attracted significant interest in nanomedicine: Drug delivery: Dendrimers provide a biocompatible shell for conjugation with drugs, antibodies, or targeting ligands, while a metal core provides controlled release and diagnostic functionality. Imaging and diagnostics: Their plasmonic and magnetic properties enhance MRI, CT, PET, and optical imaging contrast. Therapeutics: Photothermal therapy (gold-based DENs), radiosensitizers (Pt-based DENs), and antimicrobial agents (Ag-containing DENs). Biosensing: Ultra-sensitive detection of biomolecules using catalytic or electrochemical responses of bimetallic DENs.
**5. Roles in advanced nano-science and technology**
Beyond biomedicine, bimetallic DENs play a key role in - Electrocatalysis and fuel cells: Pt-based DENs show superior activity for oxygen reduction and hydrogen evolution reactions; - Environmental remediation: The degradation of pollutants via catalytic reduction or oxidation; - Energy storage and conversion: They serve as efficient catalysts in water splitting, CO_2_ reduction, and hydrogen storage systems; - Nanoelectronics and sensors: The precise control of electronic and catalytic properties enables their use in high-performance sensing devices.

**Table 6:** Expected findings of selected dendrimers and nanomaterials.

Au NPs	strong evidence for chemical detection (SERS/SPR), promising photothermal inactivation, low cytotoxicity when coated
Ag NPs	effective in antibacterial coatings and filters, with SERS utility but concerns over ecotoxicity and resistance
Se NPs	emerging as nanocarriers for drug/gene delivery with antioxidant and antimicrobial potential
Fe_3_O_4_ and oxide nanozymes (TiO_2_, CeO_2_, ZnO)	strong remediation platforms for radionuclides, chemicals, and pathogens, dependent on activation and immobilization
Dendrimers and DENs	versatile, multivalent, catalytic platforms suited for integration into devices/coatings
Fullerenes	niche but valuable for photodynamic pathogen inactivation and redox control, limited by solubility and light dependence

**Table 7:** Emerging trends to prioritize in dendrimer-based nanocomposites.

**1. Quantum dots for nuclear detection**	
Quantum dots (QDs) are gaining attention due to their potential in nuclear detection, tuneable optical properties and biocompatibility. Recent developments include the following.	
Water-Based Quantum Dot Liquid Scintillators	These offer enhanced safety and environmental benefits over traditional organic scintillators, making them suitable for applications in particle physics and nuclear detection.
Integration with Graphene	The combination of QDs with graphene has shown promise in improving the efficiency of radiation detectors, which could be beneficial for environmental monitoring and nuclear safety.
**2. Dendrimer nanocomposites in pollution sensing**	
Dendrimers, known for their uniform structure and functional groups, are being explored for their role in pollution sensing.	
Electrochemical Sensors	Dendrimer-based electrochemical sensors have demonstrated effectiveness in detecting environmental pollutants, such as heavy metals and organic contaminants.
Integration with Conductive Polymers	The combination of dendrimers with conductive polymers can enhance sensor performance, leading to more sensitive and selective detection methods.

## Data Availability

The authors declare that the data supporting the findings of this study are available within the paper.
